# Neonatal Pneumopericardium in a Nonventilated Term Infant: A Case Report and Review of the Literature

**DOI:** 10.1155/2017/3149370

**Published:** 2017-12-21

**Authors:** Smita Roychoudhury, Sharandeep Kaur, Amuchou Singh Soraisham

**Affiliations:** Section of Neonatology, Department of Pediatrics, Cumming School of Medicine, University of Calgary, Calgary, AB, Canada

## Abstract

Neonatal pneumopericardium (PPC) is a rare form of neonatal air leak syndrome with high morbidity and mortality. Air leak syndrome in the newborn is usually associated with active resuscitation, respiratory distress syndrome, meconium aspiration syndrome, mechanical ventilation, or trauma associated with labour. Neonatal PPC can be associated with other air leak syndromes such as pneumomediastinum, pneumothorax, pneumoperitoneum, and subcutaneous and interstitial emphysema. Spontaneous PPC is a rare event in the neonatal period. We report a case of PPC in association with pneumothorax in a nonventilated term infant. The infant responded to thoracocentesis without the need for pericardiocentesis.

## 1. Introduction

Pneumopericardium (PPC) in the neonatal period is a rare clinical condition, usually associated with other air leak syndrome [1–4]. It is the least frequent of the air leak syndromes and is commonly observed as a complication of mechanical ventilation, especially in premature infants with respiratory distress syndrome. It can lead to the catastrophic complication of cardiac tamponade, resulting in significant morbidity and mortality [1, 2]. In term infants, it is often seen in association with active resuscitation, meconium aspiration syndrome, or aggressive mechanical ventilation. The PPC occurring spontaneously in a nonventilated term infant is rare [5–8]. We report a case of spontaneous PPC, pneumothorax, and subcutaneous emphysema in a nonventilated term infant. The PPC was managed conservatively with a good outcome. We describe the management and review the literature.

## 2. Case Presentation

A term male infant, weighing 4130 gm, was born at 41 2/7 weeks to a healthy 34-year-old G2P0 Caucasian mother by emergency caesarean section for placental abruption. The antenatal period was unremarkable. Maternal swab for group B streptococcus was positive and received one dose of antibiotics. Membranes were ruptured for 3 hours prior to delivery, and the amniotic fluid was meconium stained. The infant was vigorous at birth and did not require any intervention. The Apgar scores were 9 and 9 at 1 and 5 minutes, respectively. The umbilical arterial pH was 7.22 with a base excess of −3. He transitioned well and was transferred to the postnatal unit with his mother for regular postnatal care. At 4 hours of age, he was noted to have respiratory distress with tachypnea and increased use of accessory muscles. He was transferred to the neonatal intensive care unit (NICU) for investigation and further management. There was no history of temperature instability, aspiration, or cyanosis.

On admission, his vital signs showed a temperature of 36.5°C, heart rate of 130 beats per minute, and respiratory rate of 90 per minute. His blood pressure was 71/41 mmHg with a mean blood pressure of 53 mmHg. The chest examination showed subcostal and intercostal retractions and diminished air entry on the right side. Cardiovascular examination showed normal heart sound, no audible murmur, and normal peripheral pulses.

In view of his respiratory distress, treatment with nasal prongs at 1 litre per minute with 30% fraction of inspired oxygen (FiO_2_) was initiated. Oxygen saturations were maintained between 90 and 95%. The first arterial blood gas on 50% FiO_2_ showed pH 7.35, CO_2_ 41, PaO_2_ 37, bicarbonate 23, and base excess of −3. Complete blood count was normal.

**Figure 1:**
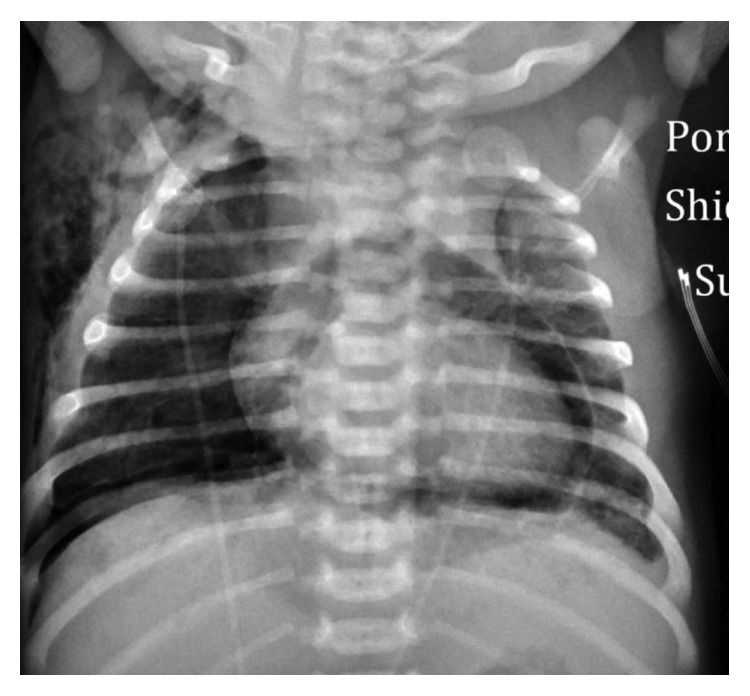
Anteroposterior chest radiograph showing pneumothorax on the right side, pneumopericardium, and subcutaneous emphysema over right axillary region.

A chest radiograph revealed a right-sided pneumothorax with a pneumopericardium and a small amount of subcutaneous emphysema in the right axilla (Figure 1). A bedside, targeted neonatal echocardiogram showed good biventricular function, nondistended inferior vena cava, and no evidence of cardiac tamponade. Because of increasing respiratory distress and increasing FiO_2_ to 70–100%, a needle aspiration of the right pneumothorax was performed under aseptic precautions, without analgesia using 23G butterfly needle attached to a syringe with a 3-way stopcock, at the right 2nd intercostal space just above the costal margin of the third rib at the midclavicular line. 16 ml of air was aspirated. FiO_2_ could be weaned down to 50% on nasal prong oxygen by the next 30 minutes. Umbilical catheters were inserted. Arterial blood gas on 50% FiO_2_ showed pH 7.40, CO_2_ 38, PaO_2_ 108, bicarbonate 24, and base excess of −1. Cardiac examination continued to be stable during and after this procedure. Respiratory distress improved after needle thoracocentesis.

Because of the risks of cardiac tamponade, the infant was transferred from the Level II NICU to the Level III NICU for observation and further management. The infant was closely observed for cardiac tamponade with cardiorespiratory monitoring and serial chest radiographs.

**Figure 2:**
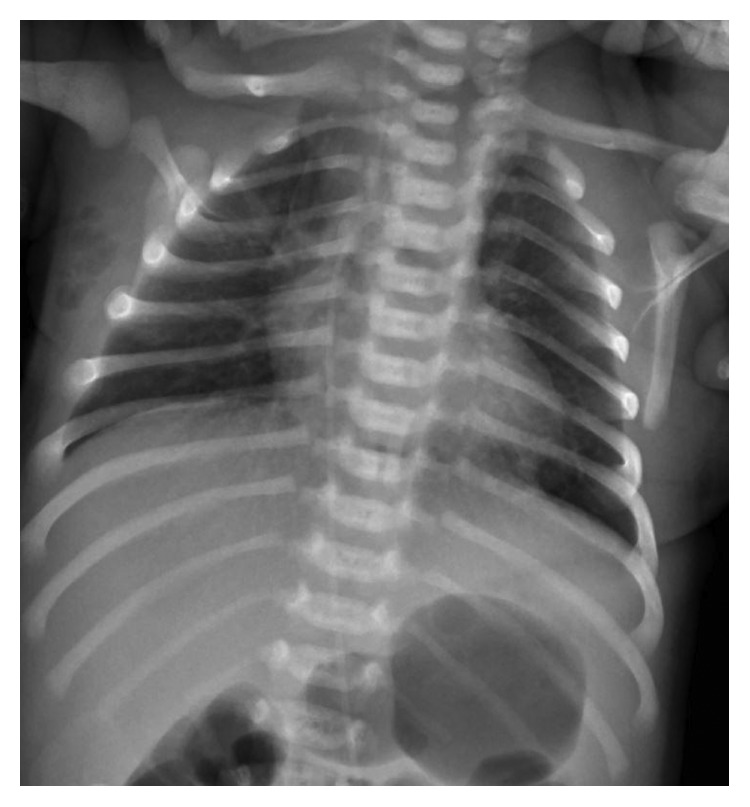
Chest radiograph on day 5 showing resolution of pneumothorax and pneumopericardium, with residual subcutaneous emphysema over right axilla.

Serial chest radiograph showed no recurrence of the pneumothorax. The PPC and subcutaneous emphysema showed improvement over the next 5 days and eventually resolved (Figure 2). His oxygen requirement was weaned gradually to room air over 48 hours, and supplemental oxygen was finally discontinued on day 3 of life. The blood culture was negative, and antibiotics were discontinued after 48 hours. The infant was breastfed by day 4 and was then discharged on day 5 of life. He has been doing well on regular follow-up with no recurrence of air leak.

## 3. Discussion

The PPC, one of the serious air leak syndrome, was first reported by Bricheteau in 1844 [9]. Between 1960 and 1990, when respiratory distress syndrome was aggressively managed by ventilation, without the use of exogenous surfactant, the incidence of air leak syndromes, characterised by pneumothorax, pneumomediastinum, PPC, pulmonary interstitial emphysema, and subcutaneous emphysema, increased significantly [2]. Factors predisposing to PPC include ongoing mechanical ventilation and continuous positive airway pressure.

The PPC can be broadly divided into two categories, namely, (a) spontaneous PPC and (b) PPC occurring in association with positive pressure ventilation. It can also occur as in isolation or in association with other forms of air leak syndrome. Spontaneous PPC, occurring in absence of mechanical ventilation, is rare in term infants. Simultaneously occurring PPC, pneumothorax, and subcutaneous emphysema are extremely rare in neonates [5, 6].

In our case, although there was meconium stained amniotic fluid, the infant was vigorous at birth. He did not require any resuscitation and was managed with routine newborn care. The respiratory distress was noted at 4 hours of age. His air leak syndrome was thought to be spontaneous in origin.

In ventilated preterm infants, PPC is usually associated with other air leaks, such as pneumothorax, pulmonary interstitial emphysema, pneumomediastinum, subcutaneous emphysema, pneumoperitoneum, and systemic vascular air embolus [10]. In the last few decades, with increasing use of antenatal steroids, the availability of exogenous surfactant therapy, and the change to gentle ventilation techniques, the incidence of air leak syndromes in general and PPC in particular has decreased significantly.

The pathogenesis of PPC has been a subject of conjecture. High bronchoalveolar pressure results in alveolar rupture and air leakage into pulmonary perivascular interstitium to the hilum and the mediastinum and then into the pericardial space at the reflection of the pulmonary veins [1–3, 11]. Rupture of this air into the pericardium has been postulated to occur in a possible anatomical area of weakness at the reflection of the parietal and visceral pericardium near the area of the pulmonary veins [7]. The pleura and pericardium share common embryological genesis, and in cases of embryological pathologies, the pleural and pericardial cavities may communicate. The mediastinal air may decrease vena caval return to the heart and compress pulmonary veins at the hilum, resulting in circulatory failure. Tension PPC inhibits cardiac contractility and venous return to the heart, resulting in decreased cardiac output.

Most theories suggest that spontaneous air leaks occur because of the sudden increase in the intrathoracic pressures within the first few breaths taken by the infant after birth. Defects in the serosal layers of the pericardium, if present, make these infants vulnerable to PPC if air ruptured into the pulmonary or mediastinal connective tissue close to the pericardium. This air, in turn, may eventually extend into the extrapleural space and cause subcutaneous emphysema. This explains why PPC is, often, associated with pneumothorax.

The clinical presentation of PPC ranges from asymptomatic to florid signs of cardiac tamponade such as hypotension, cyanosis, tachycardia, or even bradycardia, with muffled heart sounds on auscultation. The volume of pericardial air required to cause hemodynamic compromise is dependent on the rate and the amount of free air accumulation [12].

The PPC can easily be mistaken for pneumomediastinum on chest radiograph. The radiographic signs of neonatal PPC include the halo sign, that is, a continuous band of air that conforms to the cardiac outline and does not extend beyond the level of the great vessels [4]. The presence of infracardiac air confirms the diagnosis, at least in the absence of bilateral tension or central pneumothorax. The concavity of the pericardium, however, looks medially towards the midline, whereas the concavity of the pneumomediastinum is towards the peripheral border, giving the familiar “sail sign,” on a chest radiograph. A lateral chest radiograph shows air behind the sternum in pneumomediastinum while PPC shows an air shadow around the heart.

Treatment of PPC depends on the presence or absence of cardiac tamponade. Isolated asymptomatic PPC requires close monitoring for signs of cardiac tamponade. Immediate percutaneous pericardial aspiration is needed, if cardiac tamponade is suspected. In extensive or recurrent cases, a drainage tube into the pericardial sac may be required. Additional oxygen therapy as a “nitrogen washout” technique has been documented as potentially beneficial in neonatal PPC in the past [13, 14]; this approach would not be appropriate in a newborn <32 weeks' gestation at risk of possible retinopathy of prematurity. One recent study reported supplemental oxygen therapy or nitrogen washout was not associated with faster resolution of spontaneous pneumothorax [15]. These are very old practices, and we think this “nitrogen wash-out” therapy is not based on credible evidence and should be abandoned as a treatment for all air leak syndromes.

Previous studies reported poor prognosis of PPC when associated with other air leaks, especially in ventilated preterm infants [1, 11]. Hence, most authors suggested aggressive management with pericardiocentesis, even in asymptomatic cases, when associated with other air leaks. This was recommended in anticipation of clinical deterioration from cardiac tamponade, which was often fatal. Previous reports reveal that the majority of patients treated aggressively had good outcomes, while a significant number of those managed conservatively died due to the development of cardiac tamponade [1]. Our patient improved with right-sided thoracocentesis without the need for pericardiocentesis. The PPC remained asymptomatic and resolved spontaneously without invasive treatment. Conservative management with close monitoring is a reasonable choice for PPC in asymptomatic nonventilated patients. The infant should be cared for in a centre with the capability for pericardiocentesis if necessary.

To the best of our knowledge, this is the only reported case where a spontaneous PPC occurred simultaneously with other air leaks, but despite the complexity, resolution was spontaneous without need for pericardiocentesis. We reported this case to highlight the possibility of conservative management with close clinical observation and cardiorespiratory monitoring without needle pericardiocentesis in asymptomatic nonventilated term infants with spontaneous PPC.
